# Stronger Short-Term Memory, Larger Hippocampi and Area V1 in People with High VVIQ Scores

**DOI:** 10.3390/vision9030053

**Published:** 2025-07-07

**Authors:** David F. Marks

**Affiliations:** Arles, Bouches-du-Rhône, Provence-Alpes-Côte d’Azur, 13200, France; dfmarksphd@gmail.com

**Keywords:** VVIQ, visual mental imagery, short-term memory, hippocampus, cortex, individual differences, aphantasia, hyperphantasia

## Abstract

Reports of individual differences in vividness of visual mental imagery (VMI) scores raise complex questions: Are Vividness of Visual Imagery Questionnaire (VVIQ) score differences actually measuring anything? What functions do these differences serve? What is their neurological foundation? A new analysis examined visual short-term memory (VSTM) and volumes of the hippocampi, primary visual cortices, and other cortical regions among vivid and non-vivid visual imagers. In a sample of 53 volunteers aged 54 to 80 with MRI scans, the performance of ten Low VVIQ scorers was compared to that of ten High VVIQ scorers. The groups included an aphantasic with a minimum VVIQ score and a hyperphantasic with a maximum VVIQ score. The study examined volumes for 12 hippocampal subfields, 11 fields implicated in visual mental imagery including area V1 and the fusiform gyrus, and 7 motor regions. In comparison to the Low VVIQ group, High VVIQ group yielded: (i) significantly more accurate VSTM performance; and (ii) significantly larger volumes of the hippocampi and primary visual cortex. Across 47 brain regions, the average volume for the High VVIQ group exceeded that of the Low VVIQ group by 11 percent. For 47 subfields, the volumes of the hphantasic exceeded those of the aphantasic person by an average of 57 percent. Females had more accurate visual short-term memory than males and younger people were more accurate than older people. The larger visual memory capacity of females was unmatched by larger regional volume differences, which suggests that the sex difference in visual memory is caused by factors other than cortical regional size. The study confirms the existence of robust empirical associations between VMI vividness, short-term memory, regional volume of hippocampal subfields and area V1.

## 1. Introduction

In this study we are interested in the role of visual and other mental imagery in cognition, imagination, and action, directed towards the present or anticipated states of the external world. Here our focus is individual differences. This paper reports an investigation into the voluntary form of visual mental imagery (VMI) that occurs in the majority of healthy people throughout their waking lives. VMI refers to a capability within subjective experience to “see” an object, scene, or activity in the absence of the stimuli normally present when that object, scene, or activity is perceived. The well-documented, individual differences in waking VMI capacity indexed by vividness raise fundamental questions about mind–brain connections [1]. VMI vividness differences may be distinctive, but are they actually measuring anything, what functions do they serve, and what is their neurological foundation? The aim of the present study is to provide a clear answer to these three questions via an investigation of the relationship between VMI vividness scores and (1) short-term visual memory (VSTM) and (2) volumes of brain areas understood to mediate VMI.

A large body of research is consistent with the view that imagery is functionally equivalent to, but not identical to, perception showing similar anatomical patterning of neural activity in the cerebral cortex and subcortical regions, especially the hippocampus. As in perception, a preparedness for action with feelings and motives are key components of mental imagery experience [1]. Over the past 50 years much of the literature on individual differences in VMI has focused on the construct of vividness using a self-report, subjective measure called the *Vividness of Visual Imagery Questionnaire* (VVIQ) [1,2,3,4,5,6,7,8,9,10,11,12,13,14,15,16,17,18,19,20,21,22,23,24,25]. The vividness of a visual image is defined as a combination of clarity, colourfulness, and liveliness, where clarity is defined by brightness and sharpness, colourfulness by image saturation, and liveliness by vivacity, animation, feeling, solidity, projection and metamorphosis [2,3].

Mental imagery in any modality conveys intentionality—mental images are laden with vigour and feeling such as anger, anticipation, appreciation, calm, disgust, fear, or tranquillity. Not overlooking the vivid imagery of the sleep-state in dreaming, another study within this Special Issue suggests that dreams contain emotional and visual imagery vividness at significantly higher levels than is evident in waking mental imagery (Bilzer & Monzel, 2025) [4]. In everyday life, the enactive and affective attributes of mental imagery are capable of moving readers of literature to experience fictional events and characters as if they are “real”. Thus, VMI is closely associated with feeling and aesthetic appreciation of poetry, haiku, or sonnets and enhances one’s appreciation of music [5]. Recent theorizing in the General Theory of Behaviour emphasizes the role of mental imagery as a catalyst or container of feeling and motivation [6].

Here, I use an alternative methodology to present a re-analysis of an original correlational dataset from an investigation of the role of VMI in visual short-term memory (VSTM) and its foundation in brain systems, which include the subfields of the hippocampi and several other brain regions. Brain areas thought to participate in episodic memory and VMI are compared to motor control regions that, ordinarily, are not implicated in VMI functions. Previous research found that High VVIQ scorers have superior picture recall compared to Low VVIQ scorers [2,7], with females showing superior picture recall to males. The superior memory of vivid visual imagers has been independently replicated [8,9,10,11,12,13,14,15,16]. Rodway, Gillies, and Schepman (2006) [17] found high vividness participants more accurate in detecting salient changes to pictures compared to low vividness participants. An aphantasic who lacked the capacity for voluntary VMI showed worse visual working memory than the controls when high precision was required [18].

Some researchers assert that VVIQ differences are an artefact of imagers making false attributions about their capability, e.g., [19,20,21]. Yet, in spite of this criticism, experiments using the VVIQ [2,7] have repeatedly revealed robust, replicable differences between High and Low VVIQ-scoring subjects across different ages, sexes, and cultures [1,22]. The VVIQ is commonly used to identify “aphantasia” [23] and “hyperphantasia” [24], which is giving renewed impetus to the field [25]. A systematic review in this Special Issue emphasizes the need to “refine the definition and diagnosis of aphantasia” [26].

In one explanation of the distinctive individual differences in mental imagery ability, it has been suggested that the normal distribution of VMI vividness across the population may involve a subclinical form of “disconnection” [27,28,29]. This view received early support from electroencephalographic (EEG) studies that indicated a left hemisphere focus for VMI in subjects having vivid visual imagery with higher brain activation in the left parieto-occipital cortex, which was found to be lacking in matched, non-vivid imagers [30,31,32]. These EEG findings were consistent with reports of patients with brain lesions in a subfield of the posterior left hemisphere who appeared to lack the capacity of VMI generation [28,33].

Activation of the primary visual cortex is normally thought to be predictive of imagery vividness in healthy individuals [34,35,36,37,38]. However, different findings between laboratory studies with healthy volunteers and clinical case studies are reported. One clinical study found that the early visual cortex was unnecessary for VMI and, at the same time, found the left fusiform gyrus to be crucial [39]. This difference in opinion about the cortical foundation of VMI remains unresolved, although Dijkstra [40] in this Special Issue has suggested one possible solution. Bergmann, Genç, Kohler et al. (2015) [41] found a negative relationship between primary visual cortex (V1) surface area and sensory imagery strength but positive relationships between V1 and imagery precision. Other varying results have been obtained, including [42,43], who found that left precuneus activation in both static and dynamic imagery tasks was positively correlated with VVIQ scores.

Connectivity is clearly crucial. Tullo et al. (2022) [44] found the occipito-medial region has an inhibitory influence on temporal regions, and an excitatory influence of more anterior on more medial and posterior brain regions. VMI vividness was associated with connection strength from an occipital area to a parahippocampal area, especially in the left hemisphere. Ref. [45] also report increased efficiency and clustering in left inferior temporal regions in individuals with more vivid visual imagery. Thus, area V1, the left IT region, and the anterior and posterior hippocampus all appear crucial to VMI vividness [46].

The study presented here examines differences between people with Low and High VVIQ scores and gender differences in visual STM. The finding of superior visual memory of females [47] has been replicated in several contexts [48,49,50,51,52,53,54,55,56,57] while tasks involving visuospatial memory tend to favour males [58]. Relative to brain size, findings are mixed [59,60,61,62,63,64,65,66,67], suggesting that the sex difference observed on the structural level does not directly relate to sex differences in cognition, which likely depend less on how the brain is structured but on functional organization and connectivity. Dalton, Zeidman, McCormick, and Maguire (2018) [68] found that different parts of the hippocampus, together with distinct cortical regions, are recruited for scene construction or non-scene-evoking associative processing. Connections between different parts of the hippocampus and the visual–perceptual cortex appear particularly significant to the generation of VMI. Superior memory at different ages is strongly related to hippocampal structure and function [69]. Common observation and clinical testing indicate that ageing generally brings memory into decline, yet 80-year-old-plus so-called “super-agers” apparently have an episodic memory capability similar to that of 50- to 65-year-olds [70].

As noted, aphantasia refers to reduced or complete absence of voluntary VMI, while hyperphantasia refers to extremely high VMI availability and vividness [23,24]. Aphantasic subjects are selected for studies in different ways with VVIQ scores anywhere from 16 points (complete absence of visual imagery) to 32 points (“vague and dim” visual imagery, incomplete absence), e.g., [71,72]. Monzel et al. (2024) [72] investigated whether the connectivity of the hippocampus in aphantasia differs from that of controls. They investigated autobiographical memory (AM) of 14 “congenital aphantasics” and 16 controls to see how the hippocampus and visual–perceptual cortices interact during AM re-experiencing. Aphantasics showed almost no functional connectivity between the hippocampus and the visual–perceptual areas. This finding aligns with a top-down hierarchical theory of AM in which the hippocampus initiates retrieval processes in the V1 area to retrieve visual–perceptual details (Blomkvist [73]).

The impetus for the present study was a publication by Tabi et al. (2022) [74], which examined the association between VMI, short-term memory (STM), and hippocampal and primary visual cortical volumes using a correlational approach. To the best of this author’s knowledge, the study by Tabi et al. [74] is the only existing study to investigate brain regional volumes among individuals with differing VVIQ scores. In their Study 1, Tabi et al. found no evidence of a relationship between VMI and STM performance. However, significant positive correlations were found between VVIQ scores, hippocampal subfield volumes, and volumes of primary visual cortex. VSTM performance was significantly impaired in patients with AD but the VMI scores of the patients with AD were comparable to those of the age-matched elderly controls and patients with HD. Although hippocampal volumes were reduced in AD patients, there was no reduction in their VVIQ scores, suggesting that vividness may be correlated with hippocampal and visual cortex volume in healthy people but not with VSTM [74]. Here, an alternative perspective on the VVIQ/VSTM association is presented.

For this study, the dataset provided by [74] was re-analyzed using a protocol that places a predetermined number (in this case, 10) of extreme-scoring subjects into High and Low VVIQ scoring groups. Previous studies of VVIQ differences in cognition, e.g., [2,7,16] suggested that the “extreme groups” protocol would be more sensitive in detecting vividness group effects than correlational analyses conducted on an entirely unselected sample.

Another novel feature of the present study is that it is the first to investigate anatomical differences in brain regional sizes of aphantasic (aphant) and hyperphantasic (hphant) individuals.

The study tested 16 clearly stated and falsifiable hypotheses:

**H1:**  *People with vivid visual imagery have greater VSTM than people with non-vivid visual imagery.*

**H2:**  *Females have greater VSTM than males.*

**H3:**  *Younger people have greater VSTM than older people.*

**H4:**  *In brain areas related to VMI functioning (i.e., hippocampi, V1, fusiform gyrus), the High VVIQ group has larger volumes than the Low VVIQ group.*

**H5:**  *In brain areas related to VMI functioning, females have larger volumes than males.*

**H6:**  *In brain areas related to VMI functioning, younger people have larger volumes than older people.*

**H7:**  *In brain areas unrelated to VMI functioning (i.e., amygdala, Brodmann area 4), regional volume does not vary with VVIQ group.*

**H8:**  *In brain areas unrelated to VMI functioning, regional volume does not vary with gender.*

**H9:**  *In brain areas unrelated to VMI functioning, regional volume does not vary with age.*

**H10:**  *An aphant has weaker than average VSTM.*

**H11:**  *An hphant has stronger than average VSTM.*

**H12:**  *An hphant has stronger VSTM than an aphant.*

**H13:**  *An aphant has smaller than average VMI functioning volumes.*

**H14:**  *An hphant has larger than average VMI functioning volumes.*

**H15:**  *An hphant has larger VMI functioning volumes than an aphant.*

**H16:**  *Volume sizes across groups and individuals follow a predictable sequence: hphant score highest, High VVIQ group mean, entire sample mean, Low VVIQ group mean, aphant score lowest.*

## 2. Materials and Methods

The materials and methods are described in detail elsewhere [74]. (The methods, participants, data collection, and curation used in the preparation of this article were contributed by Tabi et al. (2022) [74] at the University of Oxford and are available at: https://osf.io/q37vn/ accessed on 26 June 2025. Full details are available at: https://www.sciencedirect.com/science/article/pii/S0010945221003488 accessed on 26 June 2025. Younes Adam Tabi kindly gave permission for this re-analysis but did not participate in the analyses or writing of this article.) The following is a condensed description.

### 2.1. Short-Term Memory Task

**Figure 1:**
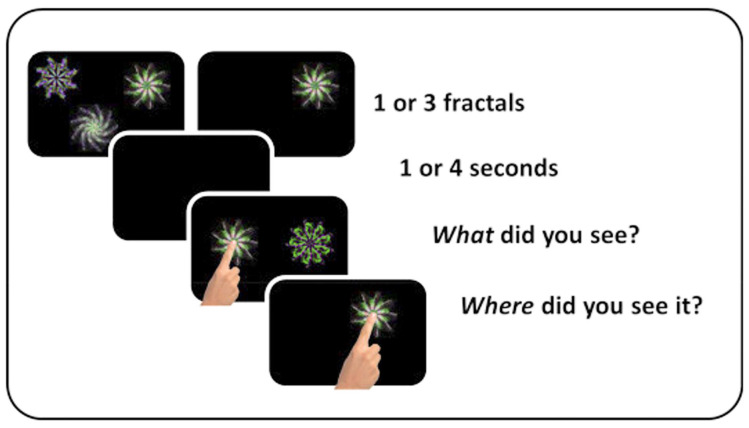
Task schematic of the visual short-term memory task. Participants saw one or three fractal designs for a period of 1 s per fractal. After a blank interval of 1 or 4 s, one of the original fractals reappeared together with a distractor fractal. Participants had to select the fractal which they thought had been previously presented (Identification accuracy) and drag it to where they recalled it had previously appeared (Localisation performance). Reproduced under a Creative Commons licence, originally published by Tabi et al. [74].

A “What was where?” object-location VSTM task (Figure 1) was used to measure VSTM [75]. At a viewing distance of approximately 30 cm, the task was performed on a touch-sensitive screen (iPad version 9.3.5 (13G36), model MGTX2B/A) with a 1536 × 2048-pixel matrix. Stimuli were drawn from a library of foils and presented on a black background, randomly selected without repetition.

Participants were presented with either one or three random shapes in the form of fractals. If one fractal was presented, it appeared for 1 s; if three fractals were presented, they appeared for 3 s. The mean duration for encoding was always 1 s per item. After a delay lasting 1 or 4 s, participants were asked to select the shape they had previously seen from two shapes presented at the centre of the screen, one of which was a never-seen foil. To select the correct shape, they were required to touch it, which provided discrete measures of Identification Accuracy and Response Time. Then they were requested to drag the selected item across the screen to its remembered location, which allowed measurement of Localisation Performance on an analogue scale: the distance between the actual location where the fractal had appeared and the location remembered by the participant.

When the person stopped dragging the chosen shape, a “Done” button appeared at the bottom centre of the screen. If participants started dragging the shape again, this disappeared and then reappeared upon release of the shape. The final position was supported by them pressing the “Done” button. To continue with the next trial, they simply pressed a “Next” button.

After 19 practice trials, the volunteers performed 120 trials over three blocks, 30 trials per condition equally distributed across blocks. The four conditions consisted of either 1 or 3 shapes combined with delays of either 1 or 4 s. The following parameters were available:

- Identification Accuracy: Trials in which the correct shape was identified out of two divided by the total number of trials of each condition. Trials in which participants did not identify the correct item were excluded from further analysis.
- Response Time: Time taken to point to the correct shape.
- Localisation Performance: Distance between the response and the original target’s location.
- Misbinding; The probability that a participant can correctly remember the appearances and locations of the shapes at test but confuses or *“misbinds”* these locations and appearances, resulting in the report of another shape’s location for a correctly identified shape.
- Guessing, Guessing indicates a random response likely given when the viewer has forgotten any precise information about the target.

For each trial, the distances between the response and (i) the target, (ii) the closest non-target, and (iii) another random trial’s non-target were compared with one another. If the distance to the target was closest, this was counted as a target response. If the distance to the non-target was closest, this was counted as a misbinding. If the distance to the random trial’s non-target was closest, this was counted as a guessing. For each trial, this procedure was repeated 5000 times with a random trial’s non-target each time. Thus, probabilities for each trial for misbinding and guessing could be calculated.

### 2.2. Vividness of Visual Imagery Questionnaire

All participants were tested on the VVIQ [2,7]. The questionnaire consists of 16 items which participants score between 1 and 5 according to how vividly they can imagine a familiar person, a shop, the sky, or a countryside scene, leading to a total score between 16 and 80.

### 2.3. MRI Analysis

A 3T Siemens Magnetom Verio syngo scanner was used to acquire T1-weighted volumetric images through a magnetization prepared rapid gradient echo protocol (MPRAGE) in sagittal orientation (TR = 2000 msec, TE = 1.94 msec, TI = 880 msec, Flip angle = 8°, FOV read = 256 mm, Voxel size = 1.0 × 1.0 × 1.0 mm). The same machine was used to record T2-FLAIR images.

FSL FIRST [76] was used to generate hippocampal and amygdala volume for both hemispheres from the T1 anatomical images. These volumes were corrected for age and total intracranial volume calculated using FSL SIENAX, which estimates total brain tissue volume from a single image, normalized for skull size. It strips non-brain tissue and uses the brain and skull images to estimate the scaling between the subject’s image and standard space. It then runs tissue segmentation to estimate the volume of brain tissue, and multiplies this by the estimated scaling factor, to reduce head size-related variability between subjects.

Grey matter volume of BA4 and V1 was calculated through the standard Freesurfer pipeline (cortical reconstruction and volumetric segmentation: http://surfer.nmr.mgh.harvard.edu/ (accessed on 1 July 2025) corrected for age and total intracranial volume. In addition, hippocampal subfields were decomposed using the software’s subfield pipeline based on T1 and T2 image inputs.

### 2.4. Availability of Data

All data are provided at: https://osf.io/q37vn/ (accessed on 1 July 2025).

### 2.5. Participants

**Figure 2:**
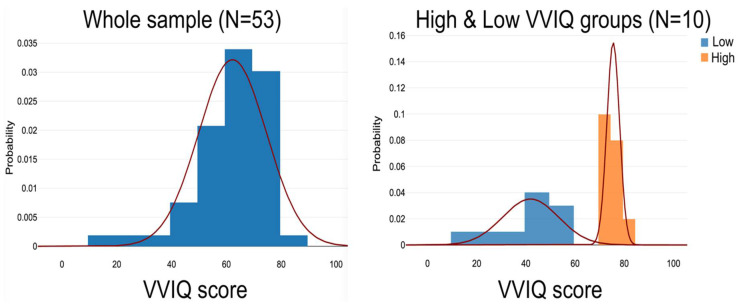
Probability distributions of the sample (left side) and the High VVIQ and Low VVIQ groups.

Participants gave their informed consent to be involved in the study, which was approved by the local ethics committee [70]. The sample consisted of 53 healthy, middle- to older-aged participants who completed an MRI scan, the VSTM task, and VVIQ. (Although Tabi et al. [74] state that their sample size was 56, the MRI dataset available at the online repository contains data for only 53 subjects.) In following the extreme groups protocol, it was decided in advance to select and compare subgroups consisting of the ten highest VVIQ and ten lowest VVIQ scorers. A gap of 21 points separated the Low VVIQ (16–52) and the High VVIQ (73–80) groups (Table 1). The High VVIQ group contained six females, one of whom scored 80 (an “hphant” who rated all 16 VVIQ items at level five—“Perfectly clear and as vivid as normal vision”). The Low VVIQ group included five females, one having a minimum score of 16 (an “aphant” who rated all 16 VVIQ items at level one—“No image at all, you only “know” that you are thinking of an object”). Figure 2 shows the probability distributions of the samples. Table 1 describes the five samples of the participants.

**Figure 3:**
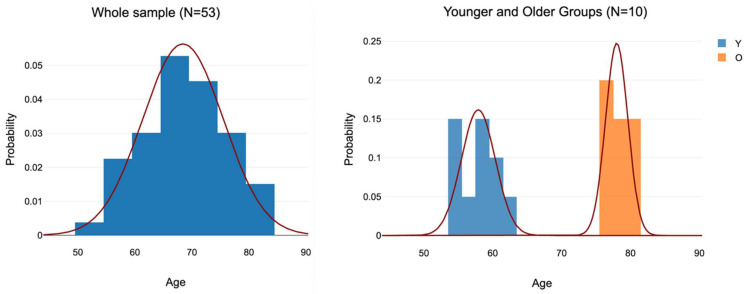
Probability distributions of the whole sample (left side) and the younger and older subgroups.

For analysis of the effect of age, the 11 oldest participants (aged 76 to 80 years) and the 10 youngest participants (aged 54 to 62) were selected, leaving an age gap of 14 years in between (Figure 3). The inclusion of three individuals tied at age 76 required N for the older group to be 11 instead of 10. The mean ages of the younger and older groups were 57.9 (SD = 2.60) and 78.0 (SD = 1.70), respectively (Figure 3; Table 1). The mean age of the original sample was 68.0 (SD = 7.2, MIN = 54, MAX = 80) of whom 29/53 (54.7%) were female.

### 2.6. Ethical Approval

All participants gave their informed consent to be involved in the study, which was approved by the local ethics committee at the University of Oxford, as reported by [74].

## 3. Results

The results for all group and individual comparisons are presented in Table S1 (Supplementary file). Normality testing of the dependent variables indicated that transformation of scores was unnecessary.

### 3.1. VVIQ Scores

Table S1 (Supplementary file) presents the mean age and VVIQ scores with standard deviations and outcomes for the members of the whole sample, the High and Low VVIQ subgroups, and the aphant and hphant individuals. The mean score difference between the Low VVIQ (41.9; SD 11.99) and High VVIQ (75.5; SD 2.72) groups was highly significant: *t*(18) = −8.6454, *p* = <0.00001, 95% confidence interval [−42.2691, −24.9309].

For the effect of gender, the sample of 53 individuals was employed. The effect of gender on VVIQ scores was slight (effect size *d* = 0.0034). A two tailed *t*-test for independent samples showed no statistical difference with t close to zero, t(51) = 0.0123, *p* = 0.99, 95%, confidence interval [−6.9707, 7.0569]. The effect size *d* was 0.0034, indicating a very small effect.

A two tailed *t*-test for independent samples showed that the difference in mean VVIQ scores between the two age groups was not statistically significant, t(18) = −1.0619, *p* = 0.302, 95%, confidence interval [−17.2756, 5.6756]. The effect size *d* of 0.4749 indicated a small effect.

### 3.2. Visual Short-Term Visual Memory (VSTM)

#### 3.2.1. Absolute Error Scores

**Figure 4:**
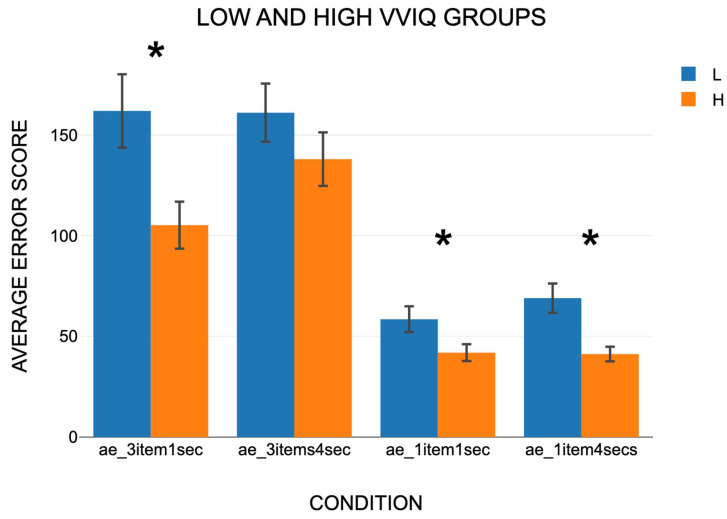
The average error scores for the Low and High VVIQ groups across the four experimental conditions of presentation of one or three shapes crossed with a 1 or 4 s delay between presentation and response. Vertical bars show one standard error. * indicates *p* < 0.05.

Four experimental conditions consisted of the presentation of one or three shapes crossed with 1 or 4 s delay between presentation and response. A two-way mixed model ANOVA showed that the difference between the four experimental conditions was statistically significant (F(3, 54) = 65.6892, *p* = <0.001) (Table S2, Supplementary file) (Figure 4). In all conditions, the Low VVIQ group produced a significantly higher absolute error score than the High VVIQ group: (F(1, 18) = 7.8919, *p* = 0.012). The interaction between VVIQ group and Condition was not significant (F(3, 54) = 1.9245, *p* = 0.137). However, the absolute error scores indicated a 38 percent lower error score for the high vividness group. The significant main effect for VVIQ group supports H1.

A two-way mixed model ANOVA with SIENAX scaling factor as a covariate showed a significant difference between females and males, F(1, 15) = 6.225, *p* = 0.025, with no interaction between gender and experimental condition, F(3, 45) = 2.457, *p* = 0.075 (Table S3, Supplementary file). This outcome supports H2.

A two-way ANOVA showed that age was significant F(1, 18) = 6.4309, *p* = 0.021, with no interaction between condition and age, F(3, 54) = 0.0814, *p* = 0.97 (Table S4, Supplementary file). This outcome supports H3.

#### 3.2.2. Mean Guessing Rates

Guessing indicates weaker memory. The High VVIQ group had lower guessing rates (*M* = 0.123, *SD* = 0.0462) than the Low VVIQ group (*M* = 0.18, *SD* = 0.0506), supporting H1 (Table S5, Supplementary file). The high vividness group had a guessing rate that was 43 percent lower than the low vividness group. A mixed model ANOVA showed that the VVIQ group difference was highly significant: F(1,18) = 11.9845, *p* = 0.003. The interaction with Condition was not significant: F(3,54) = 1.7251, *p* = 0.173. This finding supports H1.

A mixed model ANOVA showed no significant main effect between females and males, F(1, 51) = 0.7, *p* = 0.407, but there was a significant interaction, F(3, 153) = 3.2616, *p* = 0.023. In the two 3 s conditions, males had higher guessing rates than females, supporting H2 (Table S6, Supplementary file).

The younger group had lower guessing rates than the older group. A mixed model ANOVA showed that there was a significant difference between younger and older groups, F(1, 18) = 7.2431, *p* = 0.015, and a significant interaction, F(3, 54) = 4.3263, *p* = 0.008, supporting H3.

#### 3.2.3. Mean Misbinding Rates

The two misbinding conditions that produced scores (misb_3item1sec, misb_3items4sec) were analyzed in a two-way ANOVA with VVIQ group as the second factor. The High VVIQ group had significantly lower misbinding scores (*M* = 0.171, *SD* = 0.058) than the Low VVIQ group (*M* = 0.239, *SD* = 0.0816): F(1,18) = 4.5083, *p* = 0.048. There was a high/low vividness group difference on the misbinding rate of 40 percent. The interaction with Condition was not significant: F(1,18) = 3.2599. *p* = 0.088. The results support H1 (Table S7, Supplementary file).

Combining the VSTM outcomes for average error (*p* = 0.012), guessing rate (*p* = 0.003), and misbinding (*p* = 0.048) into a single *p* value using Fisher’s method gives a *p* value of 2.38 × 10^−5^.

Males made significantly more misbinding errors than females, F(1, 51) = 4.0133, *p* = 0.05, with no interaction between gender and condition, F(1, 51) = 0.0083, *p* = 0.928, supporting H2.

There was no significant difference between younger and older groups, F(1, 18) = 1.8398, *p* = 0.192, and no significant interaction between age and condition, F(1, 18) = 0.0674, *p* = 0.798, offering no support for H3.

#### 3.2.4. Correct Mean Response Times and Proportions

Neither measure produced a significant effect. Gender and age also were not significant factors for either measure; there were no significant interactions.

#### 3.2.5. Aphant and Hphant VSTM Results

Point and interval estimates of effect sizes for the aphant and hphant individuals were calculated using a Bayesian approach [77] (SingleBayes_ES.EXE). The results for the aphant and hphant were compared to those of the main sample and the two VVIQ groups (Table S1, Supplementary file). For 9/18 memory measures, the aphant showed a significantly worse memory than the average of the remaining 52 sample members. Contrary to H10, the aphant performed more strongly than the Low VVIQ group, although not significantly so. The hphant’s performance fell in line with the High VVIQ group, and was not superior to the main sample mean, contrary to H11. Although in the predicted direction, the aphant and hphant VSTM scores were not significantly different, so it did not support H12.

### 3.3. MRI Volume Scores

The 57 brain regional volumes were divided into four Sets (Table 2).

Set A consisted of 11 volume measures of four relatively large areas (at least 3000 mm^3^).

Set B consisted of 36 hippocampal subfields, 12 each for the left, right, and bilateral volumes.

Set C consisted of three volumes for the left, right, and bilateral amygdala.

Set D consisted of seven volumes for motor areas.

Sets A, B, and D were each subjected to a mixed model four-way ANOVA (IBM SPSS Version 30, Type III sum of squares) with repeated measures on Side (left, right, bilateral) and Region (12 subfields) and two independent groups factors: High vs. Low VVIQ groups and Gender. For Set C, consisting of the amygdala alone, a mixed model three-way ANOVA was conducted with repeated measures on Side (left, right, bilateral) and two independent groups factors: High vs. Low VVIQ groups and Gender.

For all four sets, the Age factor involved different samples than the VVIQ group factor and was tested in separate analyses.

#### 3.3.1. Findings for Set A Areas

**Figure 5:**
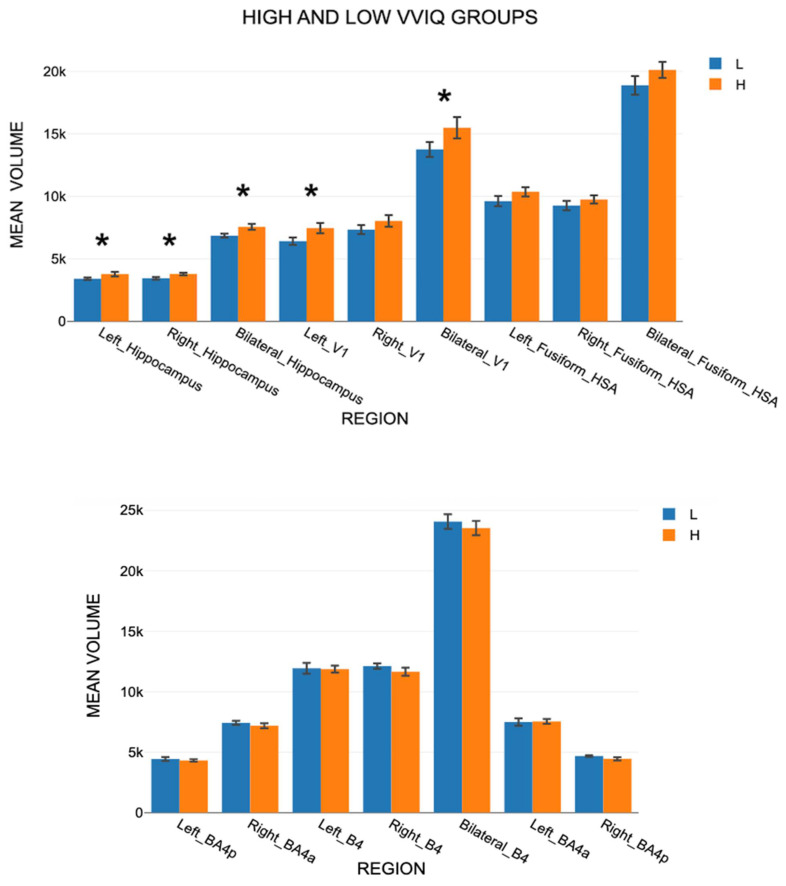
(**Upper panel**) Set A mean volumes for Low and High VVIQ groups. (**Lower panel**) Set D mean volumes for Low and High VVIQ groups. Vertical bars show one standard error. * indicates *p* < 0.05.

Cortical regional volumes were significantly higher for the High VVIQ group than for the Low VVIQ group by an average of 9.3 percent (Figure 5). An ANOVA (Table S8, Supplementary file) showed that VVIQ group was significant, F(1,16) = 7.977, *p* = 0.012, and also Gender, F(1,16) = 5.980, *p* = 0.026. VVIQ Group*Gender was not significant: F(1,16) = 0.219, *p* = 0.646. Region was significant, F(2, 32) = 237.546, *p* < 0.001, as was Side, F(2,32) = 1823.167, *p* < 0.001, and also Side*Region, F(4,64) = 169.946, *p* < 0.001. The only significant interaction between VVIQ group or Gender with Region or Side was Side*VVIQ group: F(2,32) = 4.769, *p* = 0.015. These results support H4 but not H5.

A mixed model two-way ANOVA for Set A regions with Age and Regions as factors found that neither Age, F(1, 18) = 0.1726, *p* = 0.683, nor Age*Region were significant, F(10, 180) = 0.1726, *p* = 0.683, offering no support for H6.

#### 3.3.2. Findings for Set B Subfields

**Figure 6:**
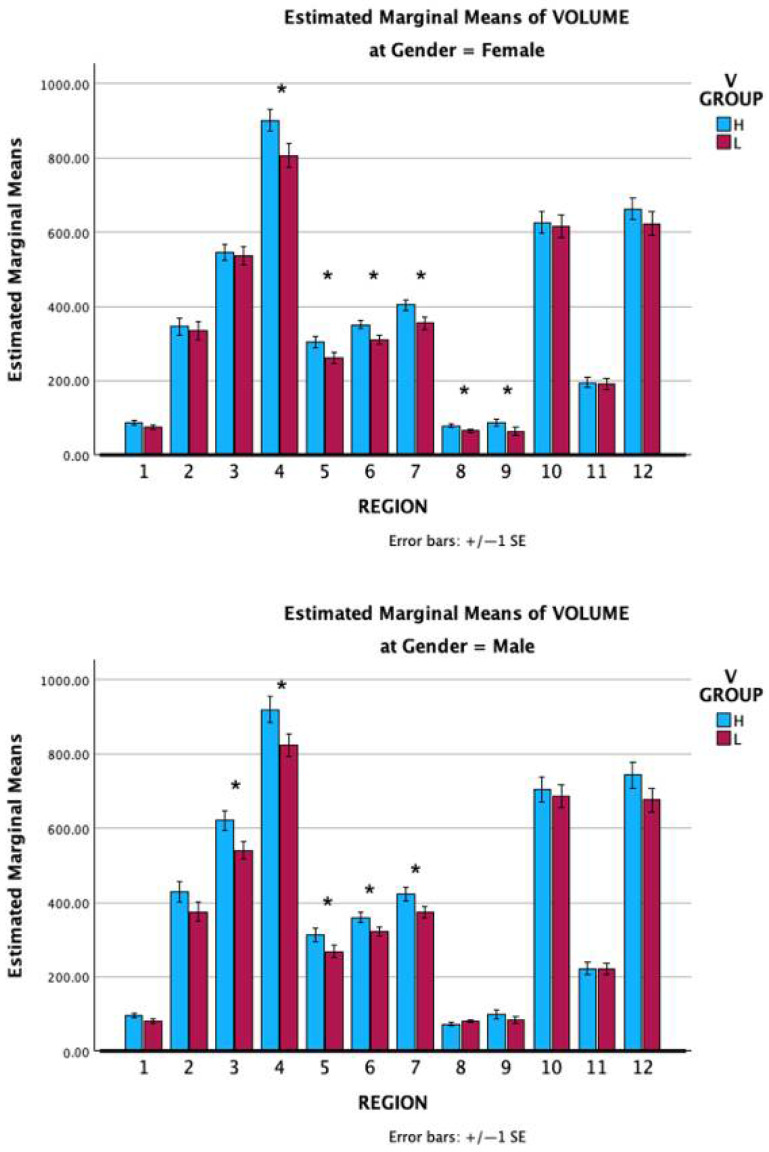
Mean hippocampal subfield volumes for Low and High VVIQ groups: upper chart, females; lower chart, males. Subfields 1–12 are as follows: 1, parasubiculum; 2, presubiculum; 3, subiculum; 4, CA1; 5, CA3; 6, CA4; 7, GC-ML-DG; 8, HATA; 9, fimbria; 10, molecular layer; 11, hippocampal fissure; 12, hippocampal tail. Vertical bars show one standard error. * indicates *p* < 0.05.

A four-way mixed model ANOVA was conducted with VVIQ group and Gender as between groups factors, and Region and Side as within-subject factors (Table S9, Supplementary file). VVIQ group was significant, F(1,16) = 8.233, *p* = 0.011 (Partial Eta Squared 0.340), as was Gender, F(1,16) = 6.252, *p* = 0.024 (Partial Eta Squared 0.281), but VVIQ group by Gender was not significant: F(1,16) = 0.156, *p* = 0.698 (Partial Eta Squared 0.010). Region was statistically significant, F(11,160) = 834.746, *p* < 0.001 (Partial Eta Squared 0.981), as was Side, F(2,32) = 3131.597, *p* < 0.001 (Partial Eta Squared 0.995), Region*Side: F (22,352) = 464.562, *p* < 0.001, and Region*Side*VVIQ group, F(22,352) = 1.622, *p* = 0.039. Side*VVIQ group was significant, F(2,32) = 6.282, *p* = 0.005, as was Side*Gender, F(2,32) = 4.807, *p* = 0.015, but Side*Gender*VVIQ group was not significant: F(2,32) = 0.151, *p* = 0.861. Region*VVIQ group was significant, F(11,352) = 2.112, *p* = 0.022, as was Region*Gender, F(11,352) = 1.968, *p* = 0.034. The mean volumes for the 12 subfields for both genders are shown in Figure 6. These findings support H4.

Sets A and B in combination contain 47 brain subregions of which 23 showed significantly higher volumes in the High than in the Low VVIQ group (Table S1, Supplementary file). The most significant were in the left hemisphere, left V1 (*p* = 0.0284), left whole hippocampus (*p* = 0.0089), left CA1 (*p* = 0.0013), left CA3 (*p* = 0.008), left CA4 (*p* = 0.0078), and the left hippocampal tail (*p* = 0.0455), all supporting H4.

Contrary to H5, male volumes for Set B exceeded female volumes. A two-way ANOVA with Age between groups and Region as a repeated measures factor showed that Region was significant, F(32, 18) = 783.606, *p* = < 0.001, but not Age, F(1,18) = 0.0109, *p* = 0.918, or Age*Region, F(32, 576) = 0.1384, *p* = 1, contrary to H6.

#### 3.3.3. Findings for the Amygdala

The effect of VVIQ group was not significant (Table S10, Supplementary file). The only significant effect was the Side*Gender interaction: F(2,32) = 4.135, *p* = 0.025. Females had significantly smaller amygdalae on the left side. A separate ANOVA found no significant Age difference and no Age*Side interaction, supporting H7–H9.

#### 3.3.4. Findings for Set D Subfields

Set D data were analyzed in a four-way ANOVA with the same independent variables as for Sets A and B with two levels of Side (left and right), as bilateral scores were unavailable for BA4a and BA4p (Table S11, Supplementary file). Region was significant, F(2, 32) = 2611.723, *p* = < 0.001, but not Side, F(1,16) = 0.051, *p* = 0.824. However, Region*Side showed significance: F(2,32) = 7.457, *p* = 0.002. Neither VVIQ group nor Gender were significant, nor was the interaction between them. The mean volumes in Set D for the two VVIQ groups were almost identical for all seven regions (Figure 5) in support of H7–H9. There was a significant Region*Gender interaction. In a separate analysis, there was no significant difference between Age groups, F(1, 18) = 0.0035, *p* = 0.954, and no interactions with Age, F(5, 90) = 0.1373, *p* = 0.983.

#### 3.3.5. Comparing VMI Active and Control Regions

The study was designed to make it possible to compare the results for regions understood to be “VMI active” with control sites such as motor regions and the amygdala that are understood to be “VMI inactive”. Table 3 presents a 2 × 2 breakdown of significant and non-significant differences obtained for the High vs. Low VVIQ group comparisons between active sites in Sets A and B and control sites in Sets C and D.

The 2 × 2 chi-squared test gave a chi-squared value (df = 1) of 8.82. *p* < 0.005 (Yates’ *p* value = 0.00886). The comparison shows that 24 significant VVIQ group differences occurred in VMI active Sets A and B only and none occurred in control Sets C and D, as hypothesized.

#### 3.3.6. Findings for Aphant and Hphant

The aphant was aged 74 and the hphant was 60. For 10 of the 47 areas in Sets A and B, the aphant yielded significantly smaller volumes than the sample average (Table 1, column 9). Also, across 47 regions in Sets A and B, the aphant yielded lower volumes (M = 1793, SD = 2281) than the hphant (M = 2172, SD = 2983). The hphant’s volumes exceeded those of the aphant by an average of 37 percent. A paired *t*-test showed that the difference was statistically significant, *t*(42) = −5.9735, *p* = < 0.001, 95% confidence interval [−225.9925, −103.2066], indicating a large effect size (d = 1.80). For only one hippocampal subfield—the left parasubiculum—the hphant yielded a smaller volume than the aphant. These findings support H15.

The aphant’s left fimbria volume of 25.23 was 1.73 standard deviations below the group average (66.67), while her right fimbria was even smaller, 10.34, 2.07 standard deviations below the group average (51.55). The aphant also had a small right amygdala of 771.01, 2.04 standard deviations below the group mean of 1310 mm^3^. The results are supportive of H13 but not at a statistically significant level.

**Figure 7:**
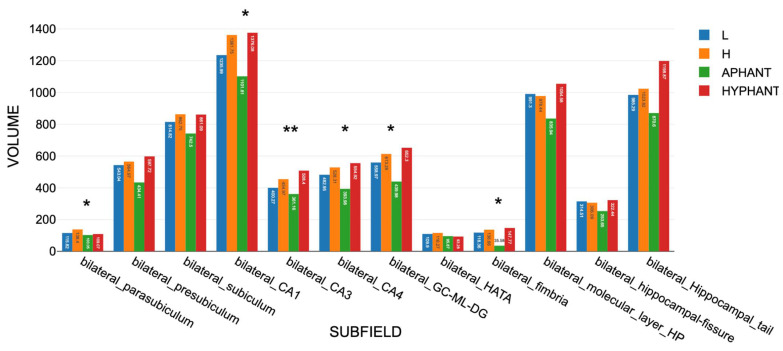
The results for the 12 bilateral hippocampal subfields, which were mirrored by the profiles for leftward and rightward volumes. * indicates *p* < 0.05; ** indicates *p* < 0.01, for the differences between the High and Low VVIQ groups.

The findings for the hphant were less significant overall. However, for the left hippocampal tail and the right fusiform gyrus, the hphant’s volume significantly exceeded that of the main sample (*p* = 0.032 and *p* = 0.0197, respectively), supporting H14. Collectively, the observed aphant/hphant differences support H15. (Figure 7)

Volumes in Set A, including area V1, showed aphant/hphant differences on a similar scale to those in Set B, supporting H15. For the motor areas in Set D, the hphant also had significantly larger volumes than the aphant: t(6) = −3.767, *p* = 0.009, d = 1.42.

#### 3.3.7. Other Analyses and Observations

For Sets A and B, there was a notable consistency in the volumes for the High and Low VVIQ groups (Table 1, columns 4 and 6). For 46 of 47 subareas, the High VVIQ had larger volumes than the Low VVIQ group, which has a probability effectively of zero, strongly supporting H4.

Another striking pattern occurred in Sets A and B (Table S1, Supplementary file, marked *). For 30 of the 57 brain regions, the mean volumes were in the predicted sequence: Hphant largest, High VVIQ group second largest, entire sample third, Low VVIQ group fourth, aphant smallest. The chance probability of this result was computed using the Binomial Theorem. There are 5! = 120 different orderings of these five categories; each occurrence of the specified ordering is a 1-in-120 event (*p* = 0.008333). In the binomial distribution, the probability of at least k successes in n trials with a probability *p* of success in any trial is given by: *P*(*x* = *k*) = (*nk*)(*p*)*k*(1 − *p*)*n* − *k*. With *n* = 57, *k* = 30, and *p* = 0.008333, the probability of obtaining at least 30 successful matches is effectively zero, providing strong support for H16.

## 4. Discussion

### 4.1. Summary of Findings

Table 4 summarises the 16 hypotheses and the study outcomes.

### 4.2. General Significance of Findings

In spite of the subjective nature of VVIQ ratings, it is notable that the predicted statistically significant differences have been found in objective measures of VSTM and brain anatomy between High and Low VVIQ scoring participants. Notably, significant VVIQ group differences only occurred in the VMI functionally active brain regions (Sets A and B) and not in the control regions (Sets C and D). The findings are extended to include significantly smaller brain region volumes between an aphantasic individual and the participant group as a whole. Pending further studies, the observed brain region volume differences could provide a helpful signpost to our understanding of VMI vividness differences within the VVIQ distribution.

#### 4.2.1. VSTM

The VSTM findings from this re-analysis of the Tabi et al. [74] dataset are substantially different from theirs. Highly significant differences between Low and High VVIQ groups on three parameters of VSTM performance support H1 that people with vivid visual imagery have greater VSTM capacity than people with non-vivid visual imagery. This result shows a substantial benefit of high VMI vividness to VSTM. The between-group differences were 38%, 43%, and 40% for absolute error, guessing, and misbinding, respectively. These three outcomes had a combined *p* value of 2.38 × 10^−5^. The higher VSTM scores obtained by females and younger participants, respectively, supported H2 that females’ VSTM would be stronger, and H3 that younger subjects’ VSTM would be stronger than the VSTM of their counterparts.

#### 4.2.2. Brain Regional Differences

The only previous study to investigate brain regional volume differences among individuals differing in VVIQ scores was by Tabi et al. [74]. Their findings of a strong association between VVIQ scores and multiple specific regional brain volumes has been fully corroborated. For the hippocampi and left V1 area, vivid visual imagers had volumes that were 16 percent larger than those for the non-vivid visual imagers. For the right V1 area, a difference of 9% failed to reach significance. The result for the left fusiform gyrus was in the predicted direction with a 7 percent difference, but it missed significance on a one-tailed test (*p* = 0.099). It is likely that larger samples would have yielded significant effects both for the right V1 area and the left fusiform gyrus.

Given the superior VSTM of females, H5 states that females would have larger hippocampal and area V1 volumes, but this was not the case—male volumes exceeded female volumes by an average of around 10 percent. This finding could have several explanations. Firstly, H5 is incorrect, and males have larger volumes than females. Second, volume is only one measure of cortical subfield size along with, for example, area and thickness. What the females lacked in volume may have been compensated for by cortical thickness, which was not measured in this study. Third, it is understood that interconnectivity between areas is an important determinant of VMI, but this was not measured. Fourth, differences in hippocampal volume depend to some degree on the system used to correct volumes for head size [78]. The SIENAX system in this study may have been imperfect for correcting volumes for gender-based head size. Fifthly, our sample sizes were small with only 11 females and 9 males, which is a major limitation.

The age differences were not significant in any volume data. The two age samples were already rather old and the age difference between the two age-based samples was relatively low, with an age gap of only 14 years (Figure 3). Evidently, this age difference was insufficient to observe brain volume differences, and/or the samples were too small, although the age effect was significant for VSTM performance, nevertheless.

#### 4.2.3. The Aphant Profile

This is the first scientific report on anatomical differences in brain region sizes of aphantasic and hyperphantasic individuals. The results show that the aphant yielded significantly smaller volumes than the entire (N = 53) sample average and had 10 significantly smaller volumes than the hphant, five of which were on the right side. The aphant had particularly small fimbria, especially on the right side. It has been reported [79] that fimbria/fornix volume is associated with spatial memory. For this sample of 53 subjects, Pearson’s r between VVIQ scores and right fimbria volumes was 0.367 (*p* < 0.01). If it had been possible, it would have been interesting to check the aphant’s olfactory sensitivity, which is also claimed to be associated with fornix volume [79]. In spite of her low volumes, the aphant scored rather well on the VSTM task with lower absolute error, guessing, and misbinding scores than the overall group means, although missing statistical significance (Table S1, Supplementary file). One may speculate that over a life of 74 years without voluntary VMI, this subject likely had developed proficient strategies for encoding memory of locations, colours, and shapes usually parts of VMI by using tags, lists, subjective probability estimates, and other symbolic processes. One may expect aphants, routinely and without effort, to be able to tag visual locations mnemonically using quick and direct labels such as “far left”, “far left and above”, “middle right, straight head”, “middle right, down a bit”, etc., along with compensatory verbal abilities and strategies deployed as “verbal scaffolding” [80].

It was notable also that the aphant’s right amygdala was considerably smaller than the group average. Together with her minute hippocampi and fimbria, one wonders whether the differences collectively contributed to her negligible capacity for VMI? As only a single aphantasic was included, caution is required when interpreting the statistical findings. Further investigation of the neurological signature of aphantasia among large samples should soon provide answers to this question.

#### 4.2.4. The Hphant Profile

The hphant’s short-term memory scores were typical of the High VVIQ group as a whole. Although 37 percent higher than the aphant’s VSTM scores, the VSTM difference was not statistically significant. Two subfields—left hippocampal tail and the right fusiform gyrus—yielded higher volumes than the entire group average and five volumes were significantly bigger than the aphant’s. The overall high VVIQ scores of the High VVIQ sample meant that the entire group was essentially hyperphantasic; their mean VVIQ score was 75.5 with a narrow SD of 2.72. Again, a more in-depth study of a larger sample is necessary.

#### 4.2.5. Consistency with Earlier EEG Findings

Prior EEG research is cited in the Introduction as implicating the left parieto-occipital cortex in mental imagery [30,31,32], which aligns with a few clinical studies [28,33,81]. This finding is reflected in the current volumetric results and appears consistent with a recent review of hemispheric asymmetries in VMI [82]. In line with {30–32}, the review [82] noted that VMI is associated with frontoparietal networks in both hemispheres; it also suggested lateralization in the temporal lobes favouring the left inferior temporal lobe for endogenous, but not electro-modulated, images.

The present finding of larger V1 volumes in High VVIQ subjects together with their larger hippocampal volumes, particularly on the left side, suggests the possibility of lateral specialization of brain function for VMI. However, contrary to that view is the lack of significance in VVIQ group differences for the left fusiform gyrus volumes. Hippocampal and brain asymmetries in VMI functioning remains a topic for further investigation.

#### 4.2.6. Relationship Between Hippocampus and Area V1

Connectivity in networks between areas acting as nodes is clearly crucial to VMI, although connectivity measures were not included in this study. Area V1 connects directly with the visual areas V2 and V4 and inferior temporal (IT) region that connect with the perirhinal and parahippocampal cortex and directly onwards via the perforant path to the posterior and anterior hippocampus. Tullo et al. (2022) [44] found that the occipito-medial region has an inhibitory influence on temporal regions and an excitatory influence of more anterior on more medial and posterior brain regions. VMI vividness was found to be associated with the connection strength from an occipital area to a parahippocampal area, especially in the left hemisphere. Kvanne et al. (2024) [45] report increased efficiency and clustering in left inferior temporal regions in individuals with more vivid visual imagery. Thus, area V1, the left IT region, and the anterior and posterior hippocampus all appear crucial to VMI vividness. In this study, the left area V1 volume and the bilateral V1 volume were larger in the High VVIQ group than in the Low VVIQ group. This finding is consistent with those of Dalton et al., 2022 [83] who used track-density imaging to examine anatomical connectivity between the cortex and the anterior–posterior axis of the in vivo human hippocampus. Their findings indicate the possibility of two medial hippocampal hubs having high anatomical connectivity: a posterior medial hub linked with visuospatial processing areas in medial parietal and occipital cortices and an anterior medial hub linked with temporopolar, inferotemporal, medial parietal, and occipital areas.

### 4.3. Strengths and Limitations

#### 4.3.1. Independence and Transparency

The data for this study were collected and curated independently of this author at the University of Oxford Medical Sciences Division, Oxford, United Kingdom. The data are shared at the Open Science Foundation website. The author had no role in the data collection or the recruitment of subjects, which were carried out entirely independently and transparently. All study data are downloadable from a public repository: https://osf.io/q37vn/ accessed on 26 June 2025.

#### 4.3.2. Measures Used

Volume is only one of several subfield size measures including also area and thickness. The females’ volume lack may have been compensated by thickness, which was not measured here. Interconnectivity between areas is another possible determinant of vivid VMI but this was also not measured. Hippocampal subfield measures depend to some degree on the correction system used to calibrate measures against head size [78]. The reliability of the SIENAX system in this study for correcting volumes for gender-based head size is an unknown, possibly confounding, factor.

A reviewer mentioned, given concerns about the accuracy of Freesurfer-derived subfields, that it would ideally have been important to clarify whether, for the original data, the authors had conducted visual inspections or carried out other quality control of the Freesurfer outputs. Unfortunately, details of error-correction with the Freesurfer-derived subfield volume scores were unavailable.

#### 4.3.3. Small Group Sizes

It has been stated that the “extreme groups” protocol is more sensitive than correlation in detecting cognitive differences between subjects varying in VMI vividness. The study protocol used here revealed a large difference in VSTM between High and Low VVIQ groups in spite of the small Ns.

The downside of the “extreme groups” protocol is the reduction in sample size from 53 to 20. Fifty-three is already not large, but the reduction to twenty, is much worse for statistical power. Substantial VVIQ differences in volumes could have overcome the relative lack of power of the small group Ns, but in the case of gender and age, the study was inconclusive in these regards.

As noted, the study included only one aphantasic and one hyperphantasic subject, and so considerable caution is warranted in interpreting the statistical findings about them.

## 5. Conclusions

(1) Are VMI-vividness differences measured with the VVIQ actually measuring anything? If we are enabled to make rich, precise, falsifiable predictions using VVIQ measures, then the answer is affirmative. VVIQ differences are measuring genuine VMI differences, and, in this study as in many others, they have produced a set of highly significant and meaningful findings.
(2) What functions do the vividness differences serve? High VMI-vividness serves three primary functions: (A) remembering recent and distant-past stimuli, scenarios, episodes, and events; (B) anticipating, foreseeing, and simulating near and distant future stimuli, scenarios, episodes, and events; and, (C) constructing phantasy for dreams, imaginary stimuli, scenarios, episodes, and events. Low or absent VMI require alternative, non-imagistic mnemonic strategies, such as scaffolding, tagging, and listing, to perform tasks but, in some cases, less efficiently.
(3) What is their neurological foundation? VMI-vividness differences are founded, at least in part, on a highly varied set of regional brain volumes which show significant, systematic associations with VVIQ scores.

## Figures and Tables

**Table 1:** The sample sizes, gender split, age means and SD, and VVIQ mean scores and SD.

Sample	N	Female: Male	Age Mean (SD)	VVIQ Mean (SD)
Whole sample	53	29:24	68.00 (7.20)	62.23 (12.53)
High VVIQ group	10	6:4	67.40 (7.83)	72.90 (9.46)
Low VVIQ group	10	5:5	68.70 (6.77)	44.50 (15.51)
Younger group (54–62 years)	10	3:7	57.9 (2.60)	58.80 (14.93)
Older group (76–80 years)	11	4:7	78.0 (1.70)	65.45 (10.55)

**Table 2:** The composition of the dataset of MRI volume scores.

Set	Left	Right	Bilateral
**A: 11 volumes**	Hippocampus	Hippocampus	Hippocampus
	Whole hippocampus	Whole hippocampus	-
	Area V1	Area V1	Area V1
	Fusiform gyrus	Fusiform gyrus	Fusiform gyrus
**B: 36 volumes**	Parasubiculum	Parasubiculum	Parasubiculum
	Presubiculum	Presubiculum	Presubiculum
	Subiculum	Subiculum	Subiculum
	CA1	CA1	CA1
	CA3	CA3	CA3
	CA4	CA4	CA4
	GC-ML-DG	GC-ML-DG	GC-ML-DG
	HATA	HATA	HATA
	Fimbria	Fimbria	Fimbria
	Molecular layer	Molecular layer	Molecular layer
	Hippocampal fissure	Hippocampal fissure	Hippocampal fissure
	Hippocampal tail	Hippocampal tail	Hippocampal tail
**C: 3 volumes**	Amygdala	Amygdala	Amygdala
**D: 7 volumes**	BA4a	BA4a	-
	BA4p	BA4p	-
	B4	B4	B4

**Table 3:** Comparison of significant VVIQ group differences obtained for VMI active and VMI inactive brain regions.

	Sets A and B VMI Active	Sets C and D VMI Inactive	Totals
**Significant VVIQ** **Group difference**	24	0	24
**Non-significant VVIQ** **Group difference**	23	10	33
**Totals**	47	10	57

**Table 4:** Summary of hypotheses and outcomes of investigation.

Label	Hypothesis	Outcome
H1	People with vivid visual imagery have greater visual short-term memory capacity than people with non-vivid visual imagery.	Strongly supported *p* < 0.001
H2	Females have greater visual short-term memory capacity than males.	Supported *p* = 0.025
H3	Younger people have greater visual short-term memory than older people.	Supported *p* = 0.01
H4	In VMIF brain regions, the High VVIQ group have larger volumes than the Low VVIQ group.	Strongly supported *p* = 0.012, *p* = 0.011, *p* = 0 (rank order correct for 46/47 areas in Sets A and B)
H5	In VMI functioning (VMIF) brain regions, females have larger volumes than males.	Unsupported
H6	In VMIF brain areas, younger people have larger volumes than older people.	Unsupported
H7	In non-VMIF brain areas, High and Low VVIQ groups have no volume differences.	Supported
H8	In non-VMIF brain areas, females and males have no volume differences.	Supported
H9	In non-VMIF brain areas, younger and older people have no volume differences.	Supported
H10	Aphant has weaker than average VSTM.	NS
H11	Hphant has stronger than average VSTM.	NS
H12	Hphant has stronger VSTM than aphant.	NS
H13	Aphant has smaller than average VMIF volumes.	Supported in 10 regions
H14	Hphant has larger than average VMIF volumes.	Supported in 2 regions
H15	Hphant has larger VMIF volumes than aphant.	Supported *p* < 0.001
H16	Volume sizes will follow a predictable sequence: hphant, High VVIQ group, entire sample mean, Low VVIQ group, aphant.	Predicted order correct 30/57 times: *p* < 0.001

## Data Availability

All data used in this study are available at: https://osf.io/q37vn/ accessed on 26 June 2025.

## Supplementary Materials

The following supporting information can be downloaded at: https://www.mdpi.com/article/10.3390/vision9030053/s1, Table S1: VVIQ scores, STVM parameters and MRI indices of subfield cortical volume for the high VVIQ group, low VVIQ group, overall sample means, hyperphantasic and aphantasic individuals; Table S2: Two-way mixed model ANOVA with VVIQ group as a between groups factor, and Condition as a repeated measures factor. Dependent variable: Absolute Error scores; Table S3: Two-way mixed model ANOVA with Gender and VVIQ group as between groups factors, and SIENAX scaling factor as a covariate. Dependent variable: Absolute Error scores; Table S4: Two-way mixed model ANOVA with Age Group as a between groups factor, and Condition as a repeated measures factor. Dependent variable: Absolute Error scores; Table S5: Two-way mixed model ANOVA with VVIQ group as a between groups factor, and Condition as a repeated measures factor. Dependent variable: Guessing scores; Table S6: Two-way mixed model ANOVA with Gender as a between groups factor, and Condition as a repeated measures factor. Dependent variable: Guessing scores; Table S7: Two-way mixed model ANOVA with VVIQ group as a between groups factor, and Condition as a repeated measures factor. Dependent variable: Mis-binding rates; Table S8: Four-way mixed model ANOVA with VVIQ group and gender as between groups factors, and region and side as within-subjects factors. Dependent variable: Set A volumes; Table S9: Four-way mixed model ANOVA with VVIQ group and gender as between groups factors, and region and side as within-subjects factors. Dependent variable: Set B volumes; Table S10: Three-way mixed model ANOVA with VVIQ group and gender as between groups factors, and side as within-subjects factors. Dependent variable: Amygdala volumes; Table S11: Four-way mixed model ANOVA with VVIQ group and gender as between groups factors, and region and side as within-subjects factors. Dependent variable: Set D volumes.
