# Supplementary material for: Stronger Short-Term Memory, Larger Hippocampi and Area V1 in People with High VVIQ Scores
Source: Vision (Basel). 2025 Jul 7;9(3):53. doi: 10.3390/vision9030053 (PMC12285986; doi:10.3390/vision9030053)
Supplement: Supplementary file 1 [file vision-09-00053-s001.zip › VISION SUPPLEMENTARY TABLE S1.pdf]

Supplementary Table S1. VVIQ scores, STVM parameters and MRI indices of subfield cortical volume for the high VVIQ group, low VVIQ group, overall sample means, hyperphantasic and aphantasic individuals

Label	Variable	Hphant N=1	High VVIQ Group Mean N=10	Sample Mean N=51	Low VVIQ Group Mean N=10	Aphant N=1	Low vs High VVIQ p	Aphant vs Entire sample p	Hphant vs Entire Sample p
D1	Age	60.00	67.8	68.42	68.300	74	NS		
C1	ACE	99.00	97.7	97.25	96.400	98	NS		
C2	VVIQ Total	80.00	75.5	62.23	41.900	16	<0.000005		
C3	Mean Absolute Error	94.125	81.558	101.300	112.628	67.745	0.0012	0.108	NS
C4	Mean Response Time	1.395	1.9168	1.881	1.978	2.07	NS	0.307	NS
C5	Mean Proportion Correct	0.916	0.945	0.928	0.925	0.93	NS	0.477	NS
C6	Mean Guessing	0.133	0.125	0.169	0.179	0.09	0.003	0.087	NS
C7	Mean Misbinding	0.152	0.171	0.229	0.239	0.12	0.048	0.070	NS
L1 *	Lt_Hippocampus	4042.6	3777.35	3600.25	3409.49	3392.32	0.044	NS	NS
L2	Lt_Amygdala	1483.4	1243.50	1299.71	1277.777	1363.48	NS	NS	NS
L3	Lt_BA4a	6295.5	7548.87	7372.94	7501.501	6502.42	NS	NS	NS
L4	Lt_BA4p	3767.3	4326.02	4220.21	4442.570	4153.33	NS	NS	NS
L5 *	Lt_V1	7646.2	7459.65	6714.83	6416.794	4956.61	0.028	0.069	NS
L6	Lt_B4	10062.8	11874.8	11593.15	11944.07	10655.7	.449	NS	NS
L7 *	Lt_Hippocampal_tail	598.059	513.214	479.08	463.736	433.840	0.0455	NS	0.0285

L8	Lt_subiculum	414.940	434.638	415.56	402.226	385.826	NS	NS	NS
L9 *	Lt_CA1	677.622	674.576	627.85	594.019	539.177	0.0013	NS	NS
L10	Lt_hippocampal-fissure	162.258	149.035	150.58	155.661	122.465	NS	NS	NS
L11 *	Lt_presubiculum	295.884	294.852	287.94	283.576	240.281	NS	NS	NS
L12	Lt_parasubiculum	48.981	70.640	63.90	62.259	58.1456	NS	NS	NS
L13 *	Lt_molecular_layer_HP	515.482	483.712	475.29	474.741	419.027	NS	NS	NS
L14 *	Lt_GC-ML-DG	311.157	292.498	279.11	262.850	211.899	NS	.01	NS
L15 *	Lt_CA3	247.694	212.428	201.71	179.530	156.281	0.0080	NS	NS
L16 *	Lt_CA4	265.4537	251.828	242.04	227.842	190.150	0.0078	0.0171	NS
L17	Lt_fimbria	51.318	70.570	67.58	63.017	25.235	NS	0. 0487	NS
L18	Lt_HATA	51.318	58.847	56.13	53.447	48.633	0.0387	NS	NS
L19 *	Lt_whole_hippocampus	3487.97	3357.809	3196.18	3067.244	2708.49	0.0089	0.0378	NS
L20*	Lt_fusiform_gyrus	11667.0	10363.2	9991.6	9620.0	8460.2	NS	NS	NS
R1	Rt_Hippocampus	3695.9	3785.83	3709.97	3447.29	3628.26	0.0163	NS	NS
R2	Rt_Amygdala	1299.9	1247.850	1320.75	1276.667	771.01	NS	0.0199	NS
R3	Rt_BA4a	6192.7	7198.073	7257.17	7432.373	6786.61	NS	NS	NS
R4	Rt_BA4p	3957.1	4461.398	4487.11	4692.102	4493.12	NS	NS	NS
R5	Rt_V1	7420.1	8033.903	7385.93	7338.094	5969.30	NS	NS	NS
R6	Rt_B4	10149.8	11659.471	11744.27	12124.475	11279.7	NS	NS	NS

R7	Rt_hippocampal_tail	600.812	528.115	507.84	510.089	436.7562	NS	NS	NS
R8 *	Rt_subiculum	446.154	427.947	419.73	405.364	356.6682	NS	NS	NS
R9 *	Rt_CA1	698.458	688.606	664.88	628.550	562.6281	0.0164	NS	NS
R10 *	Rt_hippocampal-fissure	160.184	158.692	156.31	152.767	131.182	NS	NS	NS
R11 *	Rt_presubiculum	301.831	273.392	263.78	248.605	194.130	NS	NS	NS
R12	Rt_parasubiculum	60.084	64.825	56.62	52.282	44.9054	0.0032	NS	NS
R13	Rt_molecular_layer_HP	539.100	502.341	497.41	501.027	416.914	NS	NS	NS
R14 *	Rt_GC-ML-DG	341.144	324.680	303.87	284.223	228.078	0.0026	.0027	NS
R15 *	Rt_CA3	260.705	247.887	234.00	216.831	204.874	0.0187	NS	NS
R16 *	Rt_CA4	289.4695	279.143	264.05	246.215	203.832	0.0018	.0040	NS
R17 *	Rt_fimbria	86.386	67.203	51.68	47.061	10.3436	0.0109	.0167	0.0361
R18	Rt_HATA	41.659	55.121	57.25	55.047	47.2338	NS	NS	NS
R19 *	Rt_whole_hippocampus	3666.103	3459.264	3321.10	3195.293	2706.366	0.0273	.0093	NS
R20 *	Rt_fusiform_gyrus	11881.9	9755.2	9509.5	9263.9	8874.9	NS	NS	0.0197
BIL01	Bil_Hippocampus	7738.5	7563.190	7310.22	6856.793	7020.58	0.0125	NS	NS
BIL02 *	Bil_Amygdala	2783.3	2491.353	2620.46	2554.444	2134.50	NS	NS	NS
BIL03	Bil_V1	15066.385	15493.55	14100.77	13754.888	10925.9	0.05	NS	NS
BIL04	Bil_B4	20212.6	23534.36	23337.42	24068.546	21935.4	NS	NS	NS

BIL05 *	Bil_hippocampal_tail	1198.87	1041.329	986.92	973.825	870.597	NS	NS	.0436
BIL06	Bil_subiculum	861.094	862.585	835.29	807.590	742.495	NS	NS	NS
BIL07 *	Bil_CA1	1376.08	1363.18	1292.73	1222.569	1101.80	0.0025	NS	NS
BIL08	Bil_hippocampal-fissure	322.443	307.728	306.89	308.428	253.648	NS	NS	NS
BIL09 *	Bil_presubiculum	597.716	568.246	551.71	532.180	434.412	NS	NS	NS
BIL10	Bil_parasubiculum	109.065	135.468	120.52	114.540	103.051	0.0185	NS	NS
BIL11	Bil_molecular_layer_HP	1054.582	986.054	972.69	975.768	835.942	NS	NS	NS
BIL12 *	Bil_GC-ML-DG	652.301	617.179	582.98	547.073	439.977	0.0034	0.003	NS
BIL13 *	Bil_CA3	508.400	460.316	435.71	396.361	361.155	0.0072	NS	NS
BIL14 *	Bil_CA4	554.923	530.972	506.08	474.057	393.982	0.0026	NS	NS
BIL15 *	Bil_fimbria	147.766	137.774	119.26	110.079	35.5793	0.0466	0.0206	NS
BIL16	Bil_HATA	93.277	113.969	113.38	108.495	95.867	NS	NS	NS
BIL17 *	Bil_fusiform_gyrus	23549.0	20118.4	19501.1	18883.8	17335.1	NS	NS	NS
