# Supplementary material for: Stronger Short-Term Memory, Larger Hippocampi and Area V1 in People with High VVIQ Scores
Source: Vision (Basel). 2025 Jul 7;9(3):53. doi: 10.3390/vision9030053 (PMC12285986; doi:10.3390/vision9030053)
Supplement: Supplementary file 1 [file vision-09-00053-s001.zip › VISION SUPPLEMENTARY TABLE S10.pdf]

Supplementary Table S10: Three-way mixed model ANOVA with VVIQ group and gender as between groups factors, and side as within-subjects factors. Dependent variable: Amygdala volumes.

Tests of Between-Subjects Effects

Measure: VOLUME

Transformed Variable: Average

Source	Type III Sum of Squares	df	Mean Square	F	Sig.	Partial Eta Squared	Noncent. Parameter	Observed Power[a]
Intercept	168156795.733	1	168156795.733	805.320	<0.001	0.981	805.320	1.000
Gender	400721.219	1	400721.219	1.919	0.185	0.107	1.919	0.256
VGROUP	7790.746	1	7790.746	0.037	0.849	0.002	0.037	0.054
Gender * VGROUP	9332.503	1	9332.503	0.045	0.835	0.003	0.045	0.055
Error	3340918.930	16	208807.433					

a. Computed using alpha = .05

Tests of Within-Subjects Effects

Measure: VOLUME

Source		Type III Sum of Squares	df	Mean Square	F	Sig.	Partial Eta Squared	Noncent. Parameter	Observed Power[a]
SIDE	Sphericity Assumed	21020013.838	2	10510006.919	497.534	<0.001	0.969	995.069	1.000
	Greenhouse-Geisser	21020013.838	1.891	11117156.983	497.534	<0.001	0.969	940.724	1.000
	Huynh-Feldt	21020013.838	2.000	10510006.919	497.534	<0.001	0.969	995.069	1.000
	Lower-bound	21020013.838	1.000	21020013.838	497.534	<0.001	0.969	497.534	1.000
SIDE* Gender	Sphericity Assumed	174696.830	2	87348.415	4.135	0.025	0.205	8.270	0.689
	Greenhouse-Geisser	174696.830	1.891	92394.425	4.135	0.028	0.205	7.818	0.671
	Huynh-Feldt	174696.830	2.000	87348.415	4.135	0.025	0.205	8.270	0.689

	Lower-bound	174696.830	1.000	174696.830	4.135	0.059	0.205	4.135	.481
SIDE * VGROUP	Sphericity Assumed	1541.825	2	770.913	0.036	0.964	0.002	0.073	0.055
	Greenhouse-Geisser	1541.825	1.891	815.447	0.036	0.958	0.002	0.069	0.055
	Huynh-Feldt	1541.825	2.000	770.913	0.036	0.964	0.002	0.073	0.055
	Lower-bound	1541.825	1.000	1541.825	0.036	0.851	0.002	0.036	0.054
SIDE* Gender * VGROUP	Sphericity Assumed	41201.860	2	20600.930	0.975	0.388	0.057	1.950	0.204
	Greenhouse-Geisser	41201.860	1.891	21791.020	0.975	0.384	0.057	1.844	0.199
	Huynh-Feldt	41201.860	2.000	20600.930	0.975	0.388	0.057	1.950	0.204
	Lower-bound	41201.860	1.000	41201.860	0.975	0.338	0.057	0.975	0.153
Error(SIDE)	Sphericity Assumed	675973.761	32	21124.180					
	Greenhouse-Geisser	675973.761	30.252	22344.498					
	Huynh-Feldt	675973.761	32.000	21124.180					
	Lower-bound	675973.761	16.000	42248.360					

a. Computed using alpha = .05
