# Supplementary material for: Stronger Short-Term Memory, Larger Hippocampi and Area V1 in People with High VVIQ Scores
Source: Vision (Basel). 2025 Jul 7;9(3):53. doi: 10.3390/vision9030053 (PMC12285986; doi:10.3390/vision9030053)
Supplement: Supplementary file 1 [file vision-09-00053-s001.zip › VISION SUPPLEMENTARY TABLE S11.pdf]

Supplementary Table S11: Four-way mixed model ANOVA with VVIQ group and gender as between groups factors, and region and side as within-subjects factors. Dependent variable: Set D volumes.

Tests of Between-Subjects Effects

Measure: Volume

Transformed Variable: Average

Source	Type III Sum of Squares	df	Mean Square	F	Sig.
Intercept	7412381000.380	1	7412381000.380	3539.243	<0.001
Gender	6226680.633	1	6226680.633	2.973	0.104
VGROUP	790858.401	1	790858.401	0.378	0.548
Gender * VGROUP	3012340.748	1	3012340.748	1.438	0.248
Error	33509448.853	16	2094340.553		

Tests of Within-Subjects Effects

Measure: Volume

Source		Type III Sum of Squares	df	Mean Square	F	Sig.
Region	Sphericity Assumed	1097538055.030	2	548769027.515	2611.723	<0.001
	Greenhouse-Geisser	1097538055.030	1.298	845417329.434	2611.723	<0.001
	Huynh-Feldt	1097538055.030	1.630	673321955.041	2611.723	<0.001
	Lower-bound	1097538055.030	1.000	1097538055.030	2611.723	<0.001
Region * Gender	Sphericity Assumed	1602972.111	2	801486.056	3.814	0.033
	Greenhouse-Geisser	1602972.111	1.298	1234745.707	3.814	0.055
	Huynh-Feldt	1602972.111	1.630	983397.624	3.814	0.043

	Lower-bound	1602972.111	1.000	1602972.111	3.814	0.069
Region * VGROUP	Sphericity Assumed	157383.078	2	78691.539	0.375	0.691
	Greenhouse-Geisser	157383.078	1.298	121229.857	0.375	0.602
	Huynh-Feldt	157383.078	1.630	96551.989	0.375	0.649
	Lower-bound	157383.078	1.000	157383.078	0.375	0.549
Region * Gender * VGROUP	Sphericity Assumed	445678.335	2	222839.167	1.061	0.358
	Greenhouse-Geisser	445678.335	1.298	343299.429	1.061	0.335
	Huynh-Feldt	445678.335	1.630	273416.495	1.061	0.348
	Lower-bound	445678.335	1.000	445678.335	1.061	0.318
Error(Region)	Sphericity Assumed	6723762.926	32	210117.591		
	Greenhouse-Geisser	6723762.926	20.772	323700.945		
	Huynh-Feldt	6723762.926	26.081	257807.530		
	Lower-bound	6723762.926	16.000	420235.183		
Side	Sphericity Assumed	28384.590	1	28384.590	0.051	0.824
	Greenhouse-Geisser	28384.590	1.000	28384.590	0.051	0.824
	Huynh-Feldt	28384.590	1.000	28384.590	0.051	0.824
	Lower-bound	28384.590	1.000	28384.590	0.051	0.824
Side * Gender	Sphericity Assumed	618000.896	1	618000.896	1.108	0.308
	Greenhouse-Geisser	618000.896	1.000	618000.896	1.108	0.308
	Huynh-Feldt	618000.896	1.000	618000.896	1.108	0.308

	Lower-bound	618000.896	1.000	618000.896	1.108	0.308
Side * VGROUP	Sphericity Assumed	673162.546	1	673162.546	1.207	0.288
	Greenhouse-Geisser	673162.546	1.000	673162.546	1.207	0.288
	Huynh-Feldt	673162.546	1.000	673162.546	1.207	0.288
	Lower-bound	673162.546	1.000	673162.546	1.207	0.288
Side * Gender * VGROUP	Sphericity Assumed	70706.396	1	70706.396	0.127	0.726
	Greenhouse-Geisser	70706.396	1.000	70706.396	0.127	0.726
	Huynh-Feldt	70706.396	1.000	70706.396	0.127	0.726
	Lower-bound	70706.396	1.000	70706.396	0.127	0.726
Error(Side)	Sphericity Assumed	8926023.574	16	557876.473		
	Greenhouse-Geisser	8926023.574	16.000	557876.473		
	Huynh-Feldt	8926023.574	16.000	557876.473		
	Lower-bound	8926023.574	16.000	557876.473		
Region * Side	Sphericity Assumed	803245.307	2	401622.654	7.457	0.002
	Greenhouse-Geisser	803245.307	1.537	522669.680	7.457	0.005
	Huynh-Feldt	803245.307	1.987	404279.867	7.457	0.002
	Lower-bound	803245.307	1.000	803245.307	7.457	0.015
Region * Side * Gender	Sphericity Assumed	93365.738	2	46682.869	0.867	0.430
	Greenhouse-Geisser	93365.738	1.537	60752.848	0.867	0.406
	Huynh-Feldt	93365.738	1.987	46991.732	0.867	0.429
	Lower-bound	93365.738	1.000	93365.738	0.867	0.366

Region * Side * VGROUP	Sphericity Assumed	119873.431	2	59936.716	1.113	0.341
	Greenhouse-Geisser	119873.431	1.537	78001.337	1.113	0.330
	Huynh-Feldt	119873.431	1.987	60333.269	1.113	0.341
	Lower-bound	119873.431	1.000	119873.431	1.113	0.307
Region * Side * Gender * VGROUP	Sphericity Assumed	17018.275	2	8509.137	0.158	0.855
	Greenhouse-Geisser	17018.275	1.537	11073.748	0.158	0.799
	Huynh-Feldt	17018.275	1.987	8565.436	0.158	0.853
	Lower-bound	17018.275	1.000	17018.275	0.158	0.696
Error(Region*Side)	Sphericity Assumed	1723580.494	32	53861.890		
	Greenhouse-Geisser	1723580.494	24.589	70095.590		
	Huynh-Feldt	1723580.494	31.790	54218.251		
	Lower-bound	1723580.494	16.000	107723.781		
