# Supplementary material for: Stronger Short-Term Memory, Larger Hippocampi and Area V1 in People with High VVIQ Scores
Source: Vision (Basel). 2025 Jul 7;9(3):53. doi: 10.3390/vision9030053 (PMC12285986; doi:10.3390/vision9030053)
Supplement: Supplementary file 1 [file vision-09-00053-s001.zip › VISION SUPPLEMENTARY TABLE S2.pdf]

SupplementaryTable S2: Two-way mixed model ANOVA with VVIQ group as a between groups factor, and Condition as a repeated measures factor. Dependent variable: Absolute Error scores.

	Sum of squares	df	Mean Square	F	p	η^2	η^2_p
Condition	161089.3658	3	53696.4553	65.6892	<0.001	0.5894	0.7849
V-Group	19306.2766	1	19306.2766	7.8919	0.012	0.0706	0.3048
Condition x V-Group	4719.4331	3	1573.1444	1.9245	0.137	0.0173	0.0966
Residuals (Between Subjects)	44034.2736	18	2446.3485				
Residuals (Within Subjects)	44141.3058	54	817.4316				
