# Supplementary material for: Stronger Short-Term Memory, Larger Hippocampi and Area V1 in People with High VVIQ Scores
Source: Vision (Basel). 2025 Jul 7;9(3):53. doi: 10.3390/vision9030053 (PMC12285986; doi:10.3390/vision9030053)
Supplement: Supplementary file 1 [file vision-09-00053-s001.zip › VISION SUPPLEMENTARY TABLE S3.pdf]

Supplementary Table S3: Two-way mixed model ANOVA with Gender and VVIQ group as between groups factors, and SIENAX scaling factor as a covariate. Dependent variable: Absolute Error scores.

Tests of Between-Subjects Effects

Measure: ABSOLUTE ERROR

Transformed Variable: Average

Source	Type III Sum of Squares	df	Mean Square	F	Sig.
Intercept	33.694	1	33.694	0.016	0.900
SIENAX_scalingfactor	4140.791	1	4140.791	2.005	0.177
Gender	12858.422	1	12858.422	6.225	0.025
VGROUP	9573.121	1	9573.121	4.634	0.048
Gender * VGROUP	31.351	1	31.351	0.015	0.904
Error	30985.779	15	2065.719		

Tests of Within-Subjects Effects

Measure: MEASURE_1

Source		Type III Sum of Squares	df	Mean Square	F	Sig.
CONDITION	Sphericity Assumed	456.290	3	152.097	0.185	0.906
	Greenhouse-Geisser	456.290	2.009	227.150	0.185	0.833
	Huynh-Feldt	456.290	2.939	155.278	0.185	0.902
	Lower-bound	456.290	1.000	456.290	0.185	0.673
CONDITION * SIENAX_scalingfactor	Sphericity Assumed	2708.547	3	902.849	1.101	0.359
	Greenhouse-Geisser	2708.547	2.009	1348.371	1.101	0.346
	Huynh-Feldt	2708.547	2.939	921.737	1.101	0.358
	Lower-bound	2708.547	1.000	2708.547	1.101	0.311

CONDITION * Gender	Sphericity Assumed	6043.698	3	2014.566	2.457	0.075
	Greenhouse-Geisser	6043.698	2.009	3008.677	2.457	0.102
	Huynh-Feldt	6043.698	2.939	2056.710	2.457	0.077
	Lower-bound	6043.698	1.000	6043.698	2.457	0.138
CONDITION * VGROUP	Sphericity Assumed	3437.089	3	1145.696	1.397	0.256
	Greenhouse-Geisser	3437.089	2.009	1711.054	1.397	0.263
	Huynh-Feldt	3437.089	2.939	1169.664	1.397	0.257
	Lower-bound	3437.089	1.000	3437.089	1.397	0.256
CONDITION * Gender * VGROUP	Sphericity Assumed	179.646	3	59.882	0.073	0.974
	Greenhouse-Geisser	179.646	2.009	89.432	0.073	0.930
	Huynh-Feldt	179.646	2.939	61.135	0.073	0.973
	Lower-bound	179.646	1.000	179.646	0.073	0.791
Error(CONDITION)	Sphericity Assumed	36898.269	45	819.962		
	Greenhouse-Geisser	36898.269	30.131	1224.581		
	Huynh-Feldt	36898.269	44.078	837.115		
	Lower-bound	36898.269	15.000	2459.885		
