# Supplementary material for: Stronger Short-Term Memory, Larger Hippocampi and Area V1 in People with High VVIQ Scores
Source: Vision (Basel). 2025 Jul 7;9(3):53. doi: 10.3390/vision9030053 (PMC12285986; doi:10.3390/vision9030053)
Supplement: Supplementary file 1 [file vision-09-00053-s001.zip › VISION SUPPLEMENTARY TABLE S4.pdf]

Supplementary Table S4: Two-way mixed model ANOVA with Age Group as a between groups factor, and Condition as a repeated measures factor. Dependent variable: Absolute Error scores.

	Sum of squares	df	Mean Square	F	p	η^2	η^2_p
Condition	215936.1	3	71978.703	58.8605	<0.001	0.6097	0.7658
Age Group	18929.08	1	18929.0892	6.4309	0.021	0.0534	0.2632
Condition* Age Group	298.697	3	99.5656	0.0814	0.97	0.0008	0.0045
Residuals (Between Subjects)	52982.35	18	2943.4644				
Residuals (Within Subjects)	66034.95	54	1222.8695				
