# Supplementary material for: Stronger Short-Term Memory, Larger Hippocampi and Area V1 in People with High VVIQ Scores
Source: Vision (Basel). 2025 Jul 7;9(3):53. doi: 10.3390/vision9030053 (PMC12285986; doi:10.3390/vision9030053)
Supplement: Supplementary file 1 [file vision-09-00053-s001.zip › VISION SUPPLEMENTARY TABLE S5.pdf]

Supplementary Table S5: Two-way mixed model ANOVA with VVIQ group as a between groups factor, and Condition as a repeated measures factor. Dependent variable: Guessing scores.

	Sum of squares	df	Mean Square	F	p	η^2	η^2_p
Condition	0.0331	3	0.011	15.2861	<0.001	0.1048	0.4592
VVIQ Group	0.0959	1	0.0959	11.9845	0.003	0.3038	0.3997
Cond*VVIQ Group	0.0037	3	0.0012	1.7251	0.173	0.0118	0.0875
Residuals (Between Subjects)	0.1441	18	0.008				
Residuals (Within Subjects)	0.039	54	0.0007				
