# Supplementary material for: Stronger Short-Term Memory, Larger Hippocampi and Area V1 in People with High VVIQ Scores
Source: Vision (Basel). 2025 Jul 7;9(3):53. doi: 10.3390/vision9030053 (PMC12285986; doi:10.3390/vision9030053)
Supplement: Supplementary file 1 [file vision-09-00053-s001.zip › VISION SUPPLEMENTARY TABLE S6.pdf]

Supplementary Table S6: Two-way mixed model ANOVA with Gender as a between groups factor, and Condition as a repeated measures factor. Dependent variable: Guessing scores.

	Sum of squares	df	Mean Square	F	p	η^2	η^2_p
Condition	0.1621	3	0.054	52.6698	<0.001	0.1674	0.5081
Gender	0.0087	1	0.0087	0.7	0.407	0.0089	0.0135
Condition* Gender	0.01	3	0.0033	3.2616	0.023	0.0104	0.0601
Residuals (Between Subjects)	0.6303	51	0.0124				
Residuals (Within Subjects)	0.1569	153	0.001				
