# Supplementary material for: Stronger Short-Term Memory, Larger Hippocampi and Area V1 in People with High VVIQ Scores
Source: Vision (Basel). 2025 Jul 7;9(3):53. doi: 10.3390/vision9030053 (PMC12285986; doi:10.3390/vision9030053)
Supplement: Supplementary file 1 [file vision-09-00053-s001.zip › VISION SUPPLEMENTARY TABLE S7.pdf]

Supplementary Table S7: Two-way mixed model ANOVA with VVIQ group as a between groups factor, and Condition as a repeated measures factor. Dependent variable: Mis-binding rates

	Sum of squares	df	Mean Square	F	p	η^2	η^2_p
Condition	0.0014	1	0.0014	0.2425	0.628	0.004	0.0133
VVIQ Group	0.0462	1	0.0462	4.5083	0.048	0.129	0.2003
Cond*VVIQ Group	0.0194	1	0.0194	3.2599	0.088	0.054	0.1533
Residuals (Between Subjects)	0.1846	18	0.0103				
Residuals (Within Subjects)	0.1069	18	0.0059				
