# Supplementary material for: Stronger Short-Term Memory, Larger Hippocampi and Area V1 in People with High VVIQ Scores
Source: Vision (Basel). 2025 Jul 7;9(3):53. doi: 10.3390/vision9030053 (PMC12285986; doi:10.3390/vision9030053)
Supplement: Supplementary file 1 [file vision-09-00053-s001.zip › VISION SUPPLEMENTARY TABLE S8.pdf]

Supplementary Table S8: Four-way mixed model ANOVA with VVIQ group and gender as between groups factors, and region and side as within-subjects factors. Dependent variable: Set A volumes.

Tests of Between-Subjects Effects

Measure: Volume

Transformed Variable: Average

Source	Type III Sum of Squares	df	Mean Square	F	Sig.
Intercept	108784206.906	1	108784206.906	3909.704	<0.001
VGROUP	221946.262	1	221946.262	7.977	0.012
Gender	166378.667	1	166378.667	5.980	0.026
VGROUP * Gender	6083.967	1	6083.967	0.219	0.646
Error	445186.456	16	27824.154		

Tests of Within-Subjects Effects

Source		Type III Sum of Squares	df	Mean Square	F	Sig.
Side	Sphericity Assumed	1877399304.188	2	938699652.094	1823.167	<0.001
	Greenhouse-Geisser	1877399304.188	1.499	1252273363.239	1823.167	<0.001
	Huynh-Feldt	1877399304.188	1.930	972839616.506	1823.167	<0.001
	Lower-bound	1877399304.188	1.000	1877399304.188	1823.167	<0.001
Side * Gender	Sphericity Assumed	2581732.468	2	1290866.234	2.507	0.097
	Greenhouse-Geisser	2581732.468	1.499	1722081.602	2.507	0.115
	Huynh-Feldt	2581732.468	1.930	1337814.294	2.507	0.100
	Lower-bound	2581732.468	1.000	2581732.468	2.507	0.133
Side * VGROUP	Sphericity Assumed	4911222.918	2	2455611.459	4.769	0.015

	Greenhouse-Geisser	4911222.918	1.499	3275911.325	4.769	0.026
	Huynh-Feldt	4911222.918	1.930	2544920.630	4.769	0.017
	Lower-bound	4911222.918	1.000	4911222.918	4.769	0.044
Side * Gender * VGROUP	Sphericity Assumed	462198.424	2	231099.212	0.449	0.642
	Greenhouse-Geisser	462198.424	1.499	308298.173	0.449	0.588
	Huynh-Feldt	462198.424	1.930	239504.157	.449	0.636
	Lower-bound	462198.424	1.000	462198.424	.449	0.512
Error(Side)	Sphericity Assumed	16475942.529	32	514873.204		
	Greenhouse-Geisser	16475942.529	23.987	686867.197		
	Huynh-Feldt	16475942.529	30.877	533598.845		
	Lower-bound	16475942.529	16.000	1029746.408		
Region	Sphericity Assumed	2018456727.015	2	1009228363.507	237.546	<0.001
	Greenhouse-Geisser	2018456727.015	1.536	1314058973.895	237.546	<0.001
	Huynh-Feldt	2018456727.015	1.986	1016500738.818	237.546	<0.001
	Lower-bound	2018456727.015	1.000	2018456727.015	237.546	<0.001
Region * Gender	Sphericity Assumed	5959759.638	2	2979879.819	0.701	0.503
	Greenhouse-Geisser	5959759.638	1.536	3879932.391	0.701	0.470
	Huynh-Feldt	5959759.638	1.986	3001352.466	0.701	0.502
	Lower-bound	5959759.638	1.000	5959759.638	0.701	0.415
Region * VGROUP	Sphericity Assumed	4775140.994	2	2387570.497	0.562	0.576
	Greenhouse-Geisser	4775140.994	1.536	3108720.039	0.562	0.533
	Huynh-Feldt	4775140.994	1.986	2404775.036	0.562	0.574
	Lower-bound	4775140.994	1.000	4775140.994	0.562	0.464
	Sphericity Assumed	1921316.725	2	960658.363	0.226	0.799

Region * Gender * VGROUP	Greenhouse-Geisser	1921316.725	1.536	1250818.732	0.226	0.741
	Huynh-Feldt	1921316.725	1.986	967580.748	0.226	0.797
	Lower-bound	1921316.725	1.000	1921316.725	0。226	0.641
Error(Region)	Sphericity Assumed	135953741.735	32	4248554.429		
	Greenhouse-Geisser	135953741.735	24.577	5531801.598		
	Huynh-Feldt	135953741.735	31.771	4279168.989		
	Lower-bound	135953741.735	16.000	8497108.858		
Side * Region	Sphericity Assumed	260734708.213	4	65183677.053	169.946	<0.001
	Greenhouse-Geisser	260734708.213	2.264	115186685.742	169.946	<0.001
	Huynh-Feldt	260734708.213	3.150	82769167.442	169.946	<0.001
	Lower-bound	260734708.213	1.000	260734708.213	169.946	<0.001
Side * Region * Gender	Sphericity Assumed	1094125.720	4	273531.430	0.713	0.586
	Greenhouse-Geisser	1094125.720	2.264	483359.950	.713	0.513
	Huynh-Feldt	1094125.720	3.150	347325.738	0.713	0.555
	Lower-bound	1094125.720	1.000	1094125.720	0.713	0.411
Side * Region * VGROUP	Sphericity Assumed	717350.288	4	179337.572	.468	0.759
	Greenhouse-Geisser	717350.288	2.264	316909.102	0.468	0.654
	Huynh-Feldt	717350.288	3.150	227719.917	0.468	0.715
	Lower-bound	717350.288	1.000	717350.288	0.468	0.504
Side * Region * Gender * VGROUP	Sphericity Assumed	1469378.882	4	367344.720	0.958	0.437
	Greenhouse-Geisser	1469378.882	2.264	649138.293	0.958	0.403
	Huynh-Feldt	1469378.882	3.150	466448.320	0.958	0.423
	Lower-bound	1469378.882	1.000	1469378.882	0.958	0.342
Error(Side*Region)	Sphericity Assumed	24547603.848	64	383556.310		
	Greenhouse-Geisser	24547603.848	36.217	677785.945		

	Huynh-Feldt	24547603.848	50.402	487033.532		
	Lower-bound	24547603.848	16.000	1534225.240		
