# Supplementary material for: Stronger Short-Term Memory, Larger Hippocampi and Area V1 in People with High VVIQ Scores
Source: Vision (Basel). 2025 Jul 7;9(3):53. doi: 10.3390/vision9030053 (PMC12285986; doi:10.3390/vision9030053)
Supplement: Supplementary file 1 [file vision-09-00053-s001.zip › VISION SUPPLEMENTARY TABLE S9.pdf]

Supplementary Table S9: Four-way mixed model ANOVA with VVIQ group and gender as between groups factors, and region and side as within-subjects factors. Dependent variable: Set B volumes.

Tests of Between-Subjects Effects

Measure: Volume

Transformed Variable: Average

Source	Type III Sum of Squares	df	Mean Square	F	Sig.
Intercept	108784206.906	1	108784206.906	3909.704	<0.001
VGROUP	221946.262	1	221946.262	7.977	0.012
Gender	166378.667	1	166378.667	5.980	0.026
VGROUP * Gender	6083.967	1	6083.967	0.219	0.646
Error	445186.456	16	27824.154		

Tests of Within-Subjects Effects

Measure: Volume

Source		Type III Sum of Squares	df	Mean Square	F	Sig.
Side	Sphericity Assumed	13610815.667	2	6805407.833	3007.937	<0.001
	Greenhouse-Geisser	13610815.667	1.487	9152273.210	3007.937	<0.001
	Huynh-Feldt	13610815.667	1.912	7120048.733	3007.937	<0.001
	Lower-bound	13610815.667	1.000	13610815.667	3007.937	<0.001
Side * VGROUP	Sphericity Assumed	27758.139	2	13879.069	6.134	0.006
	Greenhouse-Geisser	27758.139	1.487	18665.308	6.134	0.012
	Huynh-Feldt	27758.139	1.912	14520.754	6.134	0.006
	Lower-bound	27758.139	1.000	27758.139	6.134	0.025

Side * Gender	Sphericity Assumed	20990.811	2	10495.405	4.639	0.017
	Greenhouse-Geisser	20990.811	1.487	14114.777	4.639	0.029
	Huynh-Feldt	20990.811	1.912	10980.649	4.639	0.019
	Lower-bound	20990.811	1.000	20990.811	4.639	0.047
Side * VGROUP * Gender	Sphericity Assumed	841.323	2	420.661	0.186	0.831
	Greenhouse-Geisser	841.323	1.487	565.728	0.186	0.767
	Huynh-Feldt	841.323	1.912	440.110	0.186	0.822
	Lower-bound	841.323	1.000	841.323	0.186	0.672
Error(Side)	Sphericity Assumed	72399.462	32	2262.483		
	Greenhouse-Geisser	72399.462	23.794	3042.707		
	Huynh-Feldt	72399.462	30.586	2367.087		
	Lower-bound	72399.462	16.000	4524.966		
Region	Sphericity Assumed	37450891.110	10	3745089.111	761.547	<0.001
	Greenhouse-Geisser	37450891.110	4.121	9087818.471	761.547	<0.001
	Huynh-Feldt	37450891.110	6.770	5531948.260	761.547	<.001
	Lower-bound	37450891.110	1.000	37450891.110	761.547	<.001
Region * VGROUP	Sphericity Assumed	94596.902	10	9459.690	1.924	0.045
	Greenhouse-Geisser	94596.902	4.121	22954.847	1.924	0.115
	Huynh-Feldt	94596.902	6.770	13973.103	1.924	0.075
	Lower-bound	94596.902	1.000	94596.902	1.924	0.184
Region * Gender	Sphericity Assumed	92718.489	10	9271.849	1.885	0.051
	Greenhouse-Geisser	92718.489	4.121	22499.032	1.885	0.121
	Huynh-Feldt	92718.489	6.770	13695.639	1.885	0.081
	Lower-bound	92718.489	1.000	92718.489	1.885	0.189

Region * VGROUP * Gender	Sphericity Assumed	23192.378	10	2319.238	.472	0.906
	Greenhouse-Geisser	23192.378	4.121	5627.853	.472	0.762
	Huynh-Feldt	23192.378	6.770	3425.794	.472	0.848
	Lower-bound	23192.378	1.000	23192.378	.472	0.502
Error(Region)	Sphericity Assumed	786837.797	160	4917.736		
	Greenhouse-Geisser	786837.797	65.936	11933.359		
	Huynh-Feldt	786837.797	108.319	7264.089		
	Lower-bound	786837.797	16.000	49177.362		
Side * Region	Sphericity Assumed	4723016.514	20	236150.826	419.511	<0.001
	Greenhouse-Geisser	4723016.514	5.983	789395.147	419.511	<0.001
	Huynh-Feldt	4723016.514	11.746	402085.668	419.511	<0.001
	Lower-bound	4723016.514	1.000	4723016.514	419.511	<0.001
Side * Region * VGROUP	Sphericity Assumed	17148.118	20	857.406	1.523	0.071
	Greenhouse-Geisser	17148.118	5.983	2866.101	1.523	0.179
	Huynh-Feldt	17148.118	11.746	1459.875	1.523	0.121
	Lower-bound	17148.118	1.000	17148.118	1.523	0.235
Side * Region * Gender	Sphericity Assumed	12876.620	20	643.831	1.144	0.303
	Greenhouse-Geisser	12876.620	5.983	2152.171	1.144	0.343
	Huynh-Feldt	12876.620	11.746	1096.228	1.144	0.328
	Lower-bound	12876.620	1.000	12876.620	1.144	0.301
Side * Region * VGROUP * Gender	Sphericity Assumed	8605.442	20	430.272	0.764	0.756
	Greenhouse-Geisser	8605.442	5.983	1438.296	0.764	0.599
	Huynh-Feldt	8605.442	11.746	732.609	0.764	0.684
	Lower-bound	8605.442	1.000	8605.442	0.764	0.395
Error(Side*Region)	Sphericity Assumed	180134.318	320	562.920		

	Greenhouse-Geisser	180134.318	95.729	1881.705		
	Huynh-Feldt	180134.318	187.941	958.464		
	Lower-bound	180134.318	16.000	11258.395		
